# Enhanced Performance of Flexible Organic Photovoltaics Based on MoS_2_ Micro-Nano Array

**DOI:** 10.3390/molecules28020813

**Published:** 2023-01-13

**Authors:** Cuiyun Peng, Zhitian Ling, Minghao Qu, Chenhui Cao, Guo Chen, Wei Shi, Bin Wei

**Affiliations:** 1 School of Microelectronics and Control Engineering, Changzhou University, Changzhou 213164, China; 2 School of Mechatronic Engineering and Automation, Shanghai University, Shanghai 200072, China; 3 Key Laboratory of Advanced Display and System Application, Ministry of Education, Shanghai University, Shanghai 200072, China; 4 Anhui Sholon New Material Technology Co., Ltd. Chuzhou 239500, China

**Keywords:** organic photovoltaics, micro-nano array, MoS_2_, flexible

## Abstract

In this work, we investigated the influence of MoS_2_ functioning as an electron transport layer (ETL) on the inverted flexible organic photovoltaics (FOPVs). Three ETLs, including MoS_2_, lithium quinolate (Liq), and a MoS_2_/Liq bilayer, were evaporated onto ITO-integrated polyethylene terephthalate substrates (PET-ITO), and the properties of transmittance, water contact angle, and reflectivity of the films were analyzed. The results revealed that MoS_2_ was helpful to improve the lipophilicity of the surface of the ETL, which was conducive to the deposition of the active layer. In addition, the reflectivity of MoS_2_ to the light ranging from 400 to 600 nm was the largest among the pristine PET-ITO substrate and the PET-ITO coated with three ETLs, which promoted the efficient use of the light. The efficiency of the FOPV with MoS_2_/Liq ETL was 73% higher than that of the pristine device. This was attributed to the nearly two-fold amplification of the MoS_2_ array to the light field, which promoted the FOPV to absorb more light. Moreover, the efficiency of the FOPV with MoS_2_ was maintained under different illumination angles and bending angles. The results demonstrate the promising applications of MoS_2_ in the fabrication of FOPVs.

## 1. Introduction

Flexible organic photovoltaics (FOPVs) have become one of the hottest directions in the field of OPV research [1,2]. Neither the traditional silicon solar cells nor the emerging perovskite solar cells can be comparable with FOPV in applications requiring flexible properties. Polyethylene terephthalate (PET), which has the characteristics of light weight and bending resistance, is a widely used ideal flexible substrate in organic electronic devices, including organic field-effect transistors [3,4,5], organic light emitting diodes [6,7], and FOPVs [8,9]. The research on FOPV primarily focuses on developing new polymeric functional materials [10,11], electrodes [12,13,14,15,16,17,18], and substrates [19,20,21,22]. In addition, fabrication technology is also one of the most widely researched areas to obtain an FOPV [23,24,25,26]. Nowadays, introducing an intermediate layer between the electrode and the functional layer as an electron transport layer (ETL) to improve the performance of the FOPVs has attracted intensive attention [27,28,29,30,31].

Two-dimensional (2D) materials are a class of widely researched ETLs due to their merits of high optical transparency, high charge carrier mobility, high stability, and tunable work function [32,33,34,35]. The tunable energy gap and excellent magnetic behaviors of MoS_2_ make it one of the most attractive 2D materials in applications in ETLs [32,36,37,38,39]. Lee et al. reported an inverted OPV integrating MoS_2_ nanosheet as the ETL [40]. They revealed that the MoS_2_ nanosheet played an important role as a sub-photo sensitizer and an ETL, providing effective charge separation in the OPV. However, a deeper analysis of the performance improvement of FOPVs brought by MoS_2_ is still needed.

This work investigated the application of a MoS_2_ micro-nano array as an ETL in FOPV. Three ETLs, which are lithium quinolate (Liq), MoS_2_, and a MoS_2_/Liq bilayer, were applied in FOPV. Then the performance differences between the ETLs were studied by analyzing their transmittance, water contact angle, and reflectivity. The results revealed that MoS_2_ was helpful to improve lipophilicity, which was conducive to the deposition of the active layer. The reflectivity of MoS_2_ to the light ranging from 400 to 600 nm was the largest, which promoted the efficient use of the light. The efficiency of the FOPV with MoS_2_ EIL was 73% higher than that of the pristine FOPV. This was primarily attributed to the nearly two-fold amplification of the MoS_2_ array to the light field, which promoted the FOPV to absorb more light. Moreover, the efficiency of the FOPV with MoS_2_ was maintained under different illumination angles and bending angles. The results demonstrate the promising applications of MoS_2_ in the fabrication of FOPVs.

## 2. Results and Discussion

### 2.1. Characterization of the ETLs

The characteristics of the four kinds of films were explored in detail on the basis of transmittance, absorption, water contact angle, and reflectivity. The films containing different ETLs on the PET-ITO substrate were defined as Film A (without ETL), Film B (with Liq ETL), Film C (with MoS_2_ ETL), and Film D (with MoS_2_/Liq ETL), respectively.

**Figure 1:**
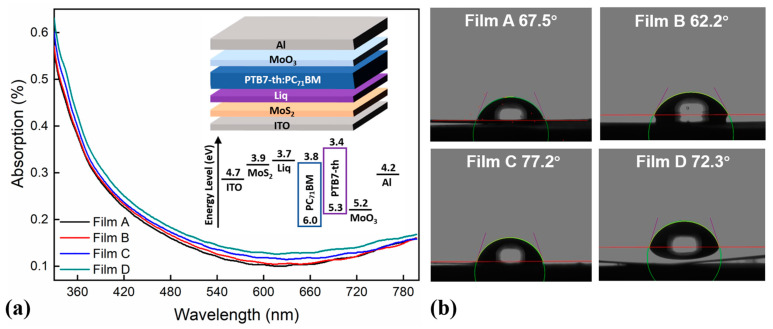
(**a**) Absorbance properties of Films A, B, C, and D; inset is the structure and energy level of Device D. (**b**) The water contact angles of Films A, B, C, and D.

Figure 1a shows the results of the absorbance properties of the four films. It can be found that if we sort the absorbance in ascending order, then the order of the films is Film A, Film B, Film C, and Film D. The transmittance calculated using the Beer–Lambert Law followed the opposite trend. Meanwhile, although the results were different from each other, there was little difference in general. It could be concluded from the results that the absorbance of the films had a slight relationship with the morphology and the structure of the film but showed a direct relationship with the thickness of the film.

Figure 1b describes the water contact angles of the four films, where it can be found that the water contact angles of Film A, Film B, Film C, and Film D are 67.5°, 62.2°, 77.2°, and 72.3°, respectively. These results indicated that Liq was helpful to improve the hydrophilicity of the film surface, while MoS_2_ was helpful to improve the lipophilicity of the film. The increased lipophilicity could decrease the surface energy difference between the ETL and the active layer [41]. Since the interfacial energy affects the thermodynamic miscibility between two adjacent layers, it is thus indicated that MoS_2_ possesses excellent miscibility with the active layer, conducive to the deposition of the active layer grown on top of the ETL.

**Figure 2:**
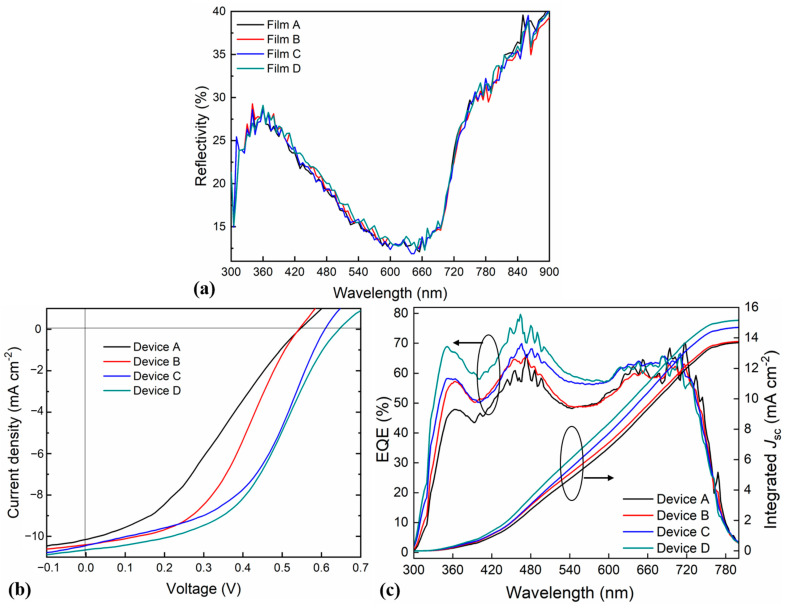
(**a**) The reflectivity test of Films A, B, C, and D. (**b**) The JV characteristic curves and (**c**) EQE and integrated J_sc_ analyses of Devices A, B, C, and D.

Figure 2a shows the reflectivity test of the four films. It can be seen from the results that the reflectivity of the four films is almost unchanged. One exception is that if the incident wavelength of the light ranges from 400 to 600 nm, the reflectivity of Film C is the largest among the four films. Here, we suppose the large reflectivity of Film C was primarily due to its nano-array morphology. The light was reflected and scattered in the arrayed structure, which led to high reflectivity. Meanwhile, the relatively thick thickness of MoS_2_ (10 nm) compared to that of Liq (3 nm) could also be the cause for its high reflectivity. In addition, there might be a waveguide function of the nano-array structure, which could further limit the transmission of light [42]. To make the active layer absorb more light, it was better if more light could be reflected by both sides of the films. Therefore, MoS_2_ was preferred as the ETL to promote the effective use of the light.

### 2.2. Effect of Difference ETLs on the Performance of FOPVs

The J-V test for four devices is shown in Figure 2b. It can be seen from Figure 2b that Device D outperforms the other three devices in terms of PCE, open-circuit voltage (V_oc_), and short-circuit current density (J_sc_). Despite that the V_oc_ and the J_sc_ of Device C are not as good as those of Device D, its filling factor (FF) is the highest among the four films. The V_oc_ and the J_sc_ of Devices A and B are nearly the same, but Device B has a larger FF than Device A. Since the work function of MoS_2_ is located between that of ITO and the LUMO of PC_71_BM, as shown in the inset of Figure 1a, the V_oc_ of Devices C and D was optimized. The corresponding values of the four devices (averaged from three devices) are summarized in Table 1. The performance of Device B is optimized compared to that of Device A, which is further increased in Device D. The V_oc_, J_sc_, FF, and PCE of Device D are 0.66 V, 10.66 mA cm^−2^, 46.71%, and 3.3%, respectively, which are the best among the four devices. The PCE of Device C and Device D increased by 63% and 73% compared to that of Device A. In addition, the J-V characteristic curves of the four devices are all “S-shaped”, which means that there exists a leakage current between layers, and hence, there still exists an opportunity for the optimization of the device structure. Meanwhile, the external quantum efficiency (EQE) and integrated J_sc_ were also analyzed. As shown in Figure 2c, the performance of Device D possessed was the best, with an EQE of over 70% at 450–500 nm and an integrated J_sc_ of 15.15 mA/cm^2^.

**Figure 3:**
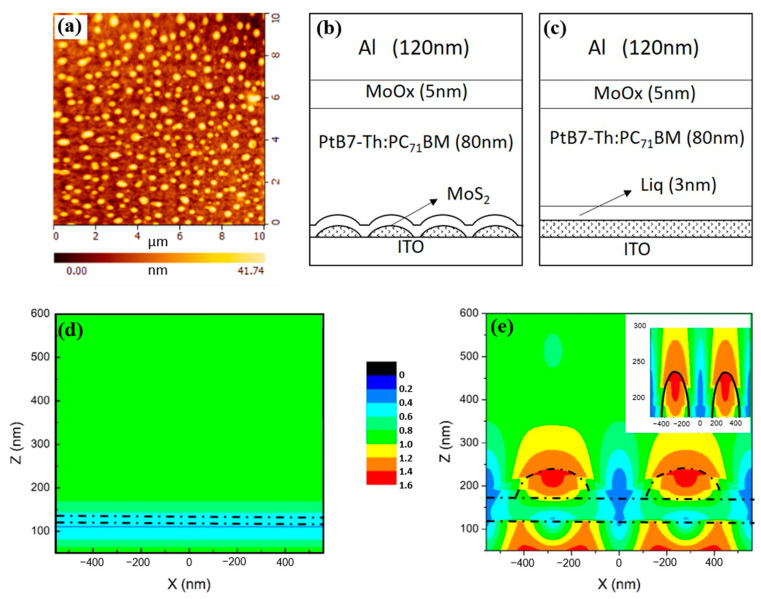
(**a**) AFM image of MoS_2_ array. Schematic diagram of the devices with (**b**) MoS_2_ array structure and (**c**) without array structure. Optical simulation of (**d**) MoS_2_ grown on PET substrate (without the nano-array structure) and (**e**) MoS_2_ grown on PET-ITO substrate (with the nano-array structure); the insets show the light intensity distribution of the film at the thickness of 175–300 nm.

From the above analyses, it could be concluded that the performance of FOPV containing MoS_2_/Liq ETL was significantly better than others. To illustrate the conclusion in detail, we analyzed the surface morphology of the MoS_2_ ETL. As depicted in Figure 3a, the MoS_2_ is no longer a thin film in terms of morphology, but rather a micro-nano array with the ability of light field amplification. The schematic diagram of the devices with the MoS_2_ array and Liq film is presented in Figure 3b,c. The micro-nano array MoS_2_ showed the optical field amplification effect, leading to the absorption of more light. To reveal the influence of the MoS_2_ array on the performance of the FOPV, we further adapted the FDTD simulation to investigate the light field of the films. As can be seen from Figure 3d,e, the amplification effect of the MoS_2_ array on the light field is significantly demonstrated. The intensity of the light field in the active layer is nearly doubled compared to the device without an array structure. This ability promoted the FOPV to absorb more light, which contributes to enhanced device performance.

### 2.3. Performance of FOPV with Different Illumination and Bending Angles

**Figure 4:**
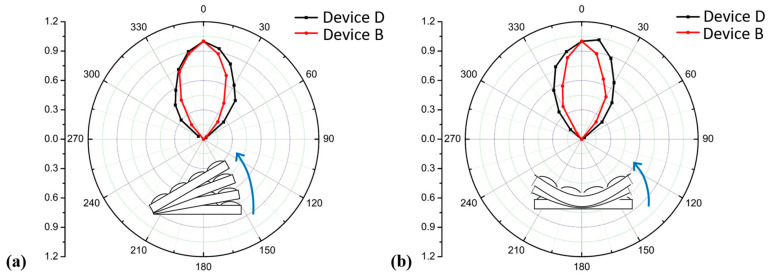
Device efficiency of Devices B and D under different (**a**) illumination angles and (**b**) bending angles.

The relationship between the efficiencies of the devices and illumination angles is shown in Figure 4a. The black line represents Device D, while the red line is Device B. The data were obtained by dividing the actual efficiency by the initial efficiency. It can be seen from Figure 4a that the efficiency of Device D decays slowly with the change in the illumination angle, which is of great significance in practical applications. The light angle sensitivity of organic photovoltaic devices seriously affects the total energy collected by the devices, while the use of a MoS_2_ array can greatly reduce the light angle sensitivity of the device, which allows the device to collect light energy more effectively at different time periods in practical applications. Therefore, the energy loss caused by the light angle was reduced, and the application of the FOPV was greatly broadened.

For FOPV, not only the illumination angle but also the bending angle plays an indispensable role in the practical application of the device, especially when the devices are applied to the surface of flexible objects such as curtains, backpacks, and solar sails, which cannot maintain a fixed angle for collecting the light source. Therefore, it is important to maintain the performance under different bending angles of the objects to which the devices are applied. Figure 4b shows the efficiency changes of the device under different bending angles. It can be seen from Figure 4b that not only is the device containing MoS_2_ insensitive to the bending angle, but its efficiency is also higher than the initial efficiency, even though the bending angle is approximately 10°. Hence, the device containing MoS_2_ was very conducive to its subsequent practical application.

## 3. Experimental Section

### 3.1. Materials and Device Fabrication

The PET-ITO substrates (30 per sheet, 150 nm) were purchased from Shanghai Quanhua Trading Co. Ltd., and the average transmittance of the substrate at 400 to 700 nm was 70%. MoS_2_ powder (98%, 5.06 g/mL at room temperature, particle size < 2 μm) and aluminum (99%) were purchased from Sigma Aldrich. Liq (99%, light yellow powder), MoO_3_ (99%, white powder), donor material PTB7-Th, and acceptor material PC_71_BM (99%, black organic material) were all purchased from Lumtec Optoelectronic Materials Co. Ltd. The proportioning method for the construction of the active layer is given here. The weight ratio of the PTB7-Th:PC71BM solvent was 2:3. The solvent was produced by mixing the chlorobenzene and the 1,8-diiodooctane according to the ratio of 97:3. The vacuum evaporation equipment used to thermally evaporate ETLs was purchased from Shenyang Lining Vacuum Co., Ltd. (Shenyang, China). MoS_2_ was placed on the tungsten boat and fixed between two electrodes in the evaporation chamber. Then, the chamber was vacuumed to 10^−4^ mbar, and the current source was turned on to evaporate the MoS_2_. Liq was put in a crucible surrounded by the heating source and was sublimated at 260−270 °C. A crystal oscillator located on the top of the chamber (controlled by the Deposition Controller, INFICON) was used to detect the rate of evaporation and the thickness of the film. We kept the lifetime of the oscillator above 85% to guarantee its high accuracy. For each ETL, we used atomic force microscope (AFM) analysis to measure their thicknesses, and the calibration parameter between the test and measured thickness was set in the Deposition Controller. Therefore, the error of the detected thickness of 10 nm (MoS_2_) and 3 nm (Liq) was less than 0.2 nm.

There are four kinds of FOPVs with different ETLs. The pristine structure was ITO (200 nm)/ETLs/PTB7-Th:PC71BM (80 nm)/MoO_3_ (5 nm)/Al (120 nm). We defined Device A as the pristine FOPV without an ETL, Device B with 3 nm Liq, Device C with 10 nm MoS_2_, and Device D with MoS_2_ (10 nm)/Liq (3 nm) as the ETL. The structure and energy level of Device D are shown in the inset of Figure 1a.

The PET-ITO substrates were ultrasonically cleaned in deionized water, acetone, and isopropanol sequentially and were dried under the tungsten lamp. Then, the substrates were UV-ozone treated for five minutes. The ETLs were thermally evaporated onto the substrate at a rate of 0.3 Å/s, and then PTB7-Th:PC71BM was spin-coated at a speed of 1000 rpm for 90 s. The spin-coated substrate was wiped with a cotton swab with chloroform to expose the electrodes. The substrate was moved into the evaporation chamber again and was vacuumized for 1 h to pump away the solvent. Then, 5 nm MoO_3_ was evaporated onto the active layer, and a 120 nm Al electrode was deposited.

### 3.2. Characterization

The FOPVs were connected to the external power supply and then continuously exposed to light with an intensity of 100 mWcm^−2^. The programmable light source meter (Keithley 2400) and the sunlight simulator AM 1.5 G were used to simulate the solar spectrum to collect the J-V characteristic curve of the device. All performance tests were carried out in the environment without encapsulated devices.

Water contact angle measurement was analyzed to reveal the wettability of the film surface by measuring the liquid–solid contact angle. In this test, the contact angle goniometer was used to measure the surface tension, wettability, and other information of deionized water and different substrates. The measurement method was a circle-fitting method.

The reflectivity test was based on the external quantum efficiency (EQE) detection platform. The 7-SCSpec solar cell measurement system manufactured by the 7-STAR company was used to first test the reflectance spectrum of the standard white plate and then test the reflectance spectrum of the sample. Eventually, the reflectivity of the sample was calculated by using the standard reflectivity.

The basic principle of the transmittance test can be described as follows: When the incident light is exposed to the surface of the sample, part of the light passing through the sample can be collected by the machine, and then the transmittance of the sample at each frequency can be obtained by comparing the wavelengths of the incident light and the collected light. The basic principle of the absorbance test is to record the absorption spectrum by taking advantage of the transition phenomenon produced when the molecules in the sample are absorbing ultraviolet and visible light. The relationship between the transmittance and the absorbance satisfies Formula (1) (Beer–Lambert Law):

(1) $$A=\mathrm{lg}\left(\frac{l}{T}\right)=Kbc$$

where *A* is the absorbance, *T* is the transmittance, *K* is the molar absorption coefficient, *b* is the sample thickness, and *c* is the sample concentration.

## 4. Conclusions

In summary, we investigated the influence of MoS_2_ ETL on the inverted flexible FOPVs. The properties of the ETLs, including transmittance, the water contact angle, and reflectivity, were systematically analyzed. The results revealed that MoS_2_ was helpful to improve the lipophilicity of the surface of the ETL, which was conducive to the deposition of the active layer. The reflectivity of MoS_2_ to light ranging from 400 to 600 nm was the largest among the pristine PET-ITO substrate and the PET-ITO coated with three ETLs, which promoted the efficient use of the light. The efficiency of the FOPV with MoS_2_ EIL was 73% higher than that of the pristine device. This was primarily attributed to the nearly two-fold amplification of the MoS_2_ array to the light field, which promoted the FOPV to absorb more light. Moreover, the efficiency of the FOPV with MoS_2_ was maintained under different illumination angles and bending angles. The results shed light on the promising applications of MoS_2_ in the fabrication of FOPVs.

## Figures and Tables

**Table 1:** The values of the four inherent parameters of the four devices.

Device	V_oc_ (V)	J_sc_ (mA cm^−2^)	FF (%)	PCE (%)
Device A	0.55 ± 0.02	10.12 ± 0.3	34.03 ± 0.1	1.89 ± 0.06
Device B	0.54 ± 0.03	10.39 ± 0.4	45.39 ± 0.2	2.54 ± 0.13
Device C	0.61 ± 0.03	10.43 ± 0.5	48.21 ± 0.2	3.07 ± 0.12
Device D	0.66 ± 0.03	10.66 ± 0.5	46.71 ± 0.2	3.29 ± 0.13

## Data Availability

Data is available from authors upon reasonable requirement.

## Sample Availability

Samples of the compounds. are available from the authors upon reasonable requirement.
